# Supplementary material for: Oral feeding with probiotic Lactobacillus rhamnosus attenuates cigarette smoke-induced COPD in C57Bl/6 mice: Relevance to inflammatory markers in human bronchial epithelial cells
Source: PLoS One. 2020 Apr 24;15(4):e0225560. doi: 10.1371/journal.pone.0225560 (PMC7182195; doi:10.1371/journal.pone.0225560)
Supplement: Attachment — Submitted filename: Response to Reviewer 2.doc [file pone.0225560.s002.doc]

**PLOS ONE**

**Responses to Reviewer**

**PONE-D-19-30819R1**

“Oral feeding with probiotic *Lactobacillus rhamnosus* attenuates cigarette smoke-induced COPD in C57Bl/6 mice: Relevance to inflammatory markers in human bronchial epithelial cells”

**Review Comments to the Author**

**Reviewer #1**

**1.** In Figure 4, neutrophil count data based on H&E staining is not convincing. There was no indication of neutrophils in the provided histology pictures. Immunostaining of neutrophils could be considered.

**R:** Dear Reviewer, we also take the opportunity to notify You that we made a mistake in Figure 4A. The correct caption of the graph is “polymorphonuclear/mm^2^ of lung tissue”. In this way, we also corrected the text exchanging “neutrophils” in the lung parenchyma for "polymorphonuclear cells". It is worth noting that this change does not compromise the characteristics of the *in vivo* COPD model, nor the interpretation nor the conclusions of results found in the present manuscript. Finally, the Reviewer is correct when affirm that to define the number of neutrophils in the lung tissue more accurately, a specific marker is needed. However, we would like to respectfully attach some manuscripts published in selective editorial policy journals where the authors evaluate the number of neutrophils in peribronchial region using the H&E staining pattern only. The manuscripts were inserted below:

- [Lanças T](https://www.ncbi.nlm.nih.gov/pubmed/?term=Lanças T%5BAuthor%5D&cauthor=true&cauthor_uid=16410372), [Kasahara DI](https://www.ncbi.nlm.nih.gov/pubmed/?term=Kasahara DI%5BAuthor%5D&cauthor=true&cauthor_uid=16410372), [Prado CM](https://www.ncbi.nlm.nih.gov/pubmed/?term=Prado CM%5BAuthor%5D&cauthor=true&cauthor_uid=16410372), [Tibério IF](https://www.ncbi.nlm.nih.gov/pubmed/?term=Tibério IF%5BAuthor%5D&cauthor=true&cauthor_uid=16410372), [Martins MA](https://www.ncbi.nlm.nih.gov/pubmed/?term=Martins MA%5BAuthor%5D&cauthor=true&cauthor_uid=16410372), [Dolhnikoff M](https://www.ncbi.nlm.nih.gov/pubmed/?term=Dolhnikoff M%5BAuthor%5D&cauthor=true&cauthor_uid=16410372). Comparison of early and late responses to antigen of sensitized guinea pig parenchymal lung strips. [**J Appl Physiol** (1985).](https://www.ncbi.nlm.nih.gov/pubmed/?term=Lanças+T+and+2006) **2006** 100(5):1610-6.

- Rigonato-Oliveira NC, Mackenzie B, Bachi ALL, Oliveira-Junior MC, Santos-Dias A, Brandao-Rangel MAR, Delle H, Costa-Guimaraes T, Damaceno-Rodrigues NR, Dulley NR, Benetti MA, Malfitano C, de Angelis C, Albertini R, Oliveira APL, Abbasi A, Northoff H, Vieira RP. [Aerobic exercise inhibits acute lung injury: from mouse to human evidence Exercise reduced lung injury markers in mouse and in cells.](https://www.ncbi.nlm.nih.gov/pubmed/29461970) **Exerc Immunol Rev. 2018**; 24:36-44.

- [Davino-Chiovatto JE](https://www.ncbi.nlm.nih.gov/pubmed/?term=Davino-Chiovatto JE%5BAuthor%5D&cauthor=true&cauthor_uid=31257011), [Oliveira-Junior MC](https://www.ncbi.nlm.nih.gov/pubmed/?term=Oliveira-Junior MC%5BAuthor%5D&cauthor=true&cauthor_uid=31257011), [MacKenzie B](https://www.ncbi.nlm.nih.gov/pubmed/?term=MacKenzie B%5BAuthor%5D&cauthor=true&cauthor_uid=31257011), [Santos-Dias A](https://www.ncbi.nlm.nih.gov/pubmed/?term=Santos-Dias A%5BAuthor%5D&cauthor=true&cauthor_uid=31257011), [Almeida-Oliveira AR](https://www.ncbi.nlm.nih.gov/pubmed/?term=Almeida-Oliveira AR%5BAuthor%5D&cauthor=true&cauthor_uid=31257011), [Aquino-Junior JCJ](https://www.ncbi.nlm.nih.gov/pubmed/?term=Aquino-Junior JCJ%5BAuthor%5D&cauthor=true&cauthor_uid=31257011), [Brito AA](https://www.ncbi.nlm.nih.gov/pubmed/?term=Brito AA%5BAuthor%5D&cauthor=true&cauthor_uid=31257011), [Rigonato-Oliveira NC](https://www.ncbi.nlm.nih.gov/pubmed/?term=Rigonato-Oliveira NC%5BAuthor%5D&cauthor=true&cauthor_uid=31257011), [Damaceno-Rodrigues NR](https://www.ncbi.nlm.nih.gov/pubmed/?term=Damaceno-Rodrigues NR%5BAuthor%5D&cauthor=true&cauthor_uid=31257011), [Oliveira APL](https://www.ncbi.nlm.nih.gov/pubmed/?term=Oliveira APL%5BAuthor%5D&cauthor=true&cauthor_uid=31257011), [Silva AP](https://www.ncbi.nlm.nih.gov/pubmed/?term=Silva AP%5BAuthor%5D&cauthor=true&cauthor_uid=31257011), [Consolim-Colombo FM](https://www.ncbi.nlm.nih.gov/pubmed/?term=Consolim-Colombo FM%5BAuthor%5D&cauthor=true&cauthor_uid=31257011), [Aimbire F](https://www.ncbi.nlm.nih.gov/pubmed/?term=Aimbire F%5BAuthor%5D&cauthor=true&cauthor_uid=31257011), [Castro-Faria-Neto HC](https://www.ncbi.nlm.nih.gov/pubmed/?term=Castro-Faria-Neto HC%5BAuthor%5D&cauthor=true&cauthor_uid=31257011), [Vieira RP](https://www.ncbi.nlm.nih.gov/pubmed/?term=Vieira RP%5BAuthor%5D&cauthor=true&cauthor_uid=31257011). Montelukast, Leukotriene Inhibitor, Reduces LPS-Induced Acute Lung Inflammation and Human Neutrophil Activation. [**Arch Bronconeumol.**](https://www.ncbi.nlm.nih.gov/pubmed/?term=Davino-Chiovatto+JE+and+2019) **2019** 55(11): 573-580.
